# Does multiple sclerosis have a zoonotic origin? Correlations with lymphocytic choriomeningitis virus infection

**DOI:** 10.3389/fimmu.2023.1217176

**Published:** 2023-06-16

**Authors:** Charissa Hogeboom

**Affiliations:** Independent, Albany, CA, United States

**Keywords:** multiple sclerosis, lymphocytic choriomeningitis virus, myeloid dendritic cells, regulatory T cells, toll-like receptor 8 (TLR8)

## 1 Introduction

Multiple sclerosis (MS) is a chronic inflammatory disease whose cause is unknown; however, a viral infection is thought to be involved. Current theory favors Epstein-Barr virus (EBV); yet EBV’s ubiquitous presence and ease of transmission are inconsistent with low MS concordance across genetically identical twins. Further, causality has not been demonstrated and the mechanism of disease induction is unknown. As an alternative hypothesis, MS may be triggered when myeloid dendritic cells (mDCs) become infected by lymphocytic choriomeningitis virus (LCMV). As mDCs are critical to thymic development of regulatory T cells (1), LCMV infection could hypothetically suggest a mechanism for disease initiation. Elucidating the mechanism of MS disease initiation is critical to our ability to prevent this debilitating disease.

## 2 Immune dysregulation in MS

MS is a chronic inflammatory disease in which self-antigens such as myelin proteins are attacked by autoreactive T cells (2). Normally, immunologic attacks on self-antigens are suppressed by a specialized subset of CD4+ cells called regulatory T cells (Tregs). However, the suppressive capacity of Tregs from relapsing-remitting MS (RRMS) patients is diminished. Peripheral Tregs from RRMS patients contain relatively few recent thymic emigrants, suggesting a defect in thymic Treg neogenesis (3).

Normal Treg development in the thymus requires strong stimulation from CD11c+ myeloid dendritic cells (mDCs). Such stimulation requires that mDCs upregulate HLA-DR and the costimulatory molecules CD40, CD80, and CD86 in response to thymic stromal lymphopoietin (TSLP) (4, 5). However, TSLP fails to induce upregulation of these costimulatory molecules in mDCs taken from RRMS patients (6). This reduced potency of TSLP is in part due to downregulation of one subunit of the TSLP receptor, IL-7Rα, independent of the IL7RA gene polymorphism (rs6897932) associated with MS (1, 6). Thus it appears that failure of self-tolerance in RRMS can be attributed, at least in part, to reduced surface molecule expression and subsequent impairment of mDCs (1).

The reason for mDC impairment is unknown but may be initiated by a viral infection. Several lines of evidence support a role for viral infection in MS. For example, MS clusters have appeared as epidemics (7) or regional hotspots (8, 9). MS relapses have appeared after respiratory infections (10). MS pathology is similar to an ongoing infectious process (11) and has been simulated experimentally through viral infection (12). And MS symptoms are relieved by administration of the antiviral cytokine, beta-interferon (IFN-β) (13).

The efficacy of IFN-β in a subset of patients points to a possible dysregulation of antiviral defense in RRMS. Normally, viral genetic material is recognized by specialized proteins called “toll-like receptors” (TLRs), which signal the release of antiviral cytokines such as type I interferons (IFN-α/β) and IL-12. This process appears to be altered in MS. Genes in the interferon pathway, such as IRF3, IRF7, and IFN, are downregulated in a subset of patients (14–16). Further, release of IL-12 in response to stimulation of TLR8, but not the other endosomal TLRs, is reduced in RRMS (17), suggesting an impairment of TLR8 signaling. Because TLR8 is expressed primarily by CD11c+ mDCs (18), the poor response to TLR8 signaling in RRMS patients may be further evidence of mDC impairment.

While the above findings are suggestive of viral involvement in RRMS, they do not tell us which virus(es) may initiate disease, nor the mechanism by which they do it. One highly researched candidate is Epstein-Barr virus (EBV). An association between EBV and MS was hypothesized based on several factors, such as higher seropositivity against EBV among MS patients (19–21); higher presence of EBV in MS brain (19, 22, 23); an increase in EBV-specific CD8+ cells during MS relapses (19); and EBV-specific oligoclonal bands in MS CSF (24). However, a causal relationship between EBV and MS has not been demonstrated. It is unclear how EBV, which infects B cells, could cause the impairment of mDCs or the diminished response to TLR8 stimulation observed in RRMS patients. Nor does EBV explain the geographic distribution of MS. For example, EBV’s high overall prevalence (~95%) and ease of person-to-person transmission is inconsistent with the low MS concordance across monozygotic twin pairs (25–30). Further, MS prevalence follows a latitudinal gradient, with increasing risk farther from the equator (31); in contrast, exposure to EBV is delayed in countries of higher latitude (32), forming a reverse latitudinal gradient. This inconsistency has been rationalized by assuming the “hygiene hypothesis,” which proposes that delayed exposure to EBV increases risk of MS. However, the hygiene hypothesis would lead to the untenable conclusion that EBV-negative individuals incur the highest MS risk (33). Recent evidence suggests that EBV may be a marker of chronic inflammation, indicating T cell exhaustion and an inability to clear the virus (34, 35), rather than a causative agent per se.

Given the downregulation of costimulatory molecules and receptors on mDCs in RRMS, impairing their ability to stimulate Treg development in the thymus, it is reasonable to hypothesize that a trigger virus for MS may impair mDCs. One such virus is lymphocytic choriomeningitis virus (LCMV).

## 3 LCMV: a hypothesis

LCMV is a zoonotic ssRNA virus whose natural host is the common house mouse. Transmission to humans occurs primarily by inhalation of aerosolized rodent excreta, by bites, or by contact with rodent urine, feces, or saliva (36). LCMV infection in humans is usually mild or asymptomatic, but may occasionally lead to aseptic meningitis (37).

LCMV strains differ with respect to tropism and pathogenicity. While the wild-type strain induces acute infection that is rapidly cleared, strains carrying the F260L mutation in the GP1 glycoprotein gene suppress the immune response and establish persistent infection (38). The F260L variant infects humans^1^ as well as mice. This variant preferentially infects CD11c+ mDCs (38), the cell type that is impaired in RRMS.

Any pathogen proposed as an instigator of MS should explain how mDCs become impaired. LCMV may provide some answers in this respect. LCMV persistent strains preferentially infect CD11c+ mDCs, resulting in downregulation of key cell surface molecules involved in antigen presentation and T cell maturation (39). Specifically, expression of MHC (HLA in humans), CD40, CD80, and CD86 is reduced in LCMV-infected mDCs. As a consequence, infected mDCs bind developing T cells less tightly and fail to stimulate their proliferation (39). Such persistent viral infection mimics the impairments observed in RRMS patients (**Table 1**). Contributing to the Treg failure in RRMS is downregulation of the IL-7Rα subunit on both T cells and mDCs (6). While the IL-7Rα subunit was not explicitly studied in mDCs from persistently infected mice, LCMV infection reduced expression of IL-7R on T cells (41, 42). The similarities between LCMV infection and RRMS with respect to cell surface molecule expression on mDCs and, possibly, T cells is intriguing and worthy of further investigation.

**Table 1:** Parallels between RRMS and persistent LCMV infection.

RRMS Characteristic	References	LCMV Characteristic	References
Downregulation of CD40, CD80, CD86, HLA-DR on CD11c+ mDCs	(1)	Downregulation of CD40, CD80, CD86, MHC class I & II on CD11c+ mDCs infected with persistent LCMV strain	(39, 40)
Impaired ability of CD11c+ mDCs to stimulate effector T cells	(1)	Impaired ability of LCMV-infected CD11c+ mDCs to stimulate effector T cells	(39, 40)
Downregulation of IL-7Rα subunit on CD11c+ mDCs and T cells	(6)	Downregulation of IL-7R on LCMV-infected T cells	(41, 42)
Reduced release of IL-12 in response to stimulation of TLR8, but normal response to TLR7 stimulation. (TLR8 is expressed by mDCs, while TLR7 is expressed by pDCs.)	(17)	Reduced release of IL-12 from LCMV-infected mDCs	(40, 43–45)
IFN-β is effective in subset of patients, whose pre-treatment expression of IFN-β genes is downregulated	(14, 15)	LCMV inhibits transcription of IFN-β genes	(46, 47)
MS prevalence is <1% globally	(48)	LCMV prevalence is <5% globally	(49, 50)
MS concordance across monozygotic twins is low (~25%)	(25–30)	LCMV is not spread person-to-person	(36, 50)
Geographically most prevalent in the equatorial zone globally with latitudinal gradient	(31)	Reported LCMV prevalence in humans and mice all fall within the equatorial zone and show a latitudinal gradient	(49)
Croatia was country with highest MS incidence in 2005–2007	(48)	LCMV seroprevalence in Croatia was 36% in 2006 (compare to <5% globally)	(51)
Slovenia was country with second-highest MS prevalence in 2005–2007; high MS prevalence along Croatia/Slovenia border	(8, 48)	LCMV seroprevalence among mice in Slovenia and Croatia was 47% (compare to <5% globally)	(52)
Motif for MBP peptide-HLA binding and recognition by autoreactive T cells from MS patients is VHFFK	(53)	Best match IHFYR by sequence homology comes from LMCV nucleoprotein	(49)

A pathogen involved in initiating MS should also explain the observed dysregulation of the innate immune system. Two aspects of innate immunity altered in RRMS are relevant here. First, TLR8 is less effective than other endosomal TLRs in signaling the release of IL-12 from PBMCs of RRMS patients (17). Second, a subset of RRMS patients who benefit from exogenous IFN-β treatment show downregulation of genes in the interferon pathway (14, 54). Both these aspects of innate immunity can be impacted by LCMV. TLR8 senses ssRNA and is expressed primarily on mDCs, the cell type infected by LCMV persistent strains. This contrasts with TLR7, which also senses ssRNA but is expressed by a different cell type (plasmacytoid DCs). The difference between TLR8 and TLR7 signaling in RRMS patients suggests a defect in mDCs, which could hypothetically be a consequence of LCMV infection. LCMV infection does inhibit IL-12 secretion (40, 43–45), although the involvement of TLR8 has not been tested directly. Further, LCMV inhibits IFN production by blocking activation of the interferon transcription factor IRF3 (46, 47). The predilection of LCMV to infect the primary cell type expressing TLR8, along with its ability to suppress IL-12 and IFN release, are consistent with similar observations from RRMS patients (**Table 1**). LCMV-induced inhibition of cytokine release and evasion of host recognition should be investigated further to determine whether they contribute to immune dysfunction in MS.

Finally, a pathogen involved in MS initiation should be consistent with epidemiologic observations about MS. Epidemiology was one of the earliest tools used to study MS. While MS research has now moved more toward molecular and genetic epidemiology, some of the old population-based findings still hold. MS continues to be more prevalent in the temperate zone geographically, with a latitudinal gradient (31). MS prevalence is still under 1% (48, 55), and concordance across monozygotic twin pairs remains around 1 in 4 (25–30). These characteristics cannot easily be explained by EBV, which is highly prevalent, easily spread person-to-person, and shows a reverse latitudinal gradient of childhood exposure. In contrast, LCMV is most prevalent in the temperate zone with a latitudinal gradient (49) and overall prevalence on the order of 2–5% (49, 50). The virus is transmitted directly from rodents and their excreta, without human-to-human spread (50), consistent with the low disease concordance reported by MS twin studies. Aside from these global measures, a few regional hotspots of MS are approximately colocalized with areas of high LCMV prevalence (**Table 1**). For example, the country with the highest MS incidence during the reporting period 2005–2007 was Croatia (48); at the same time, Croatia’s Vir Island reported that 36% of residents were positive for anti-LCMV antibodies (51). While the evidence is circumstantial, the low prevalence, temperate zone distribution, and lack of human-to-human transmission that characterize LCMV are consistent with the low twin concordance and geographic distribution observed for MS.

## 4 Conclusion

The infectious pathogen that induces MS has not yet been identified with certainty. LCMV is a viable candidate due to its ability to impair mDCs, whose function is required for thymic development of regulatory T cells. However, the evidence in favor of LCMV, such as immune evasion or geographic distribution, is largely circumstantial and does not constitute proof. We believe that rigorous scientific evidence, either for or against the LCMV hypothesis, is important and feasible to obtain.

A preliminary assessment can be both simple and cost-effective. Since seroprevalence of anti-LCMV antibodies in the US and Western Europe is low, on the order of 5%, testing for increased seroprevalence among MS patients could be accomplished with a very small sample of subjects.

A study evaluating the association between a pathogen and MS should consider the impact of gene-environment interactions. The genetic influence most likely relevant to pathogen-induced autoimmunity is HLA type. A pathogenic virus may initiate inflammation by mimicking an endogenous peptide when bound to HLA; however, the orientation of any given peptide will vary by HLA type (56–58). For example, an immunodominant peptide from myelin basic protein (MBP) binds to the high-risk HLA DRB1*1501 in a different orientation than it does to other HLA types (57), where it may not bind at all. A peptide from LCMV predicted to mimic this MBP peptide (49) meets criteria (53) for binding to HLA DRB1*1501, but would likely not match criteria for binding to another HLA type. It is plausible, perhaps likely, that any specific virus operating through molecular mimicry may be successful in only a subset of the population. For these reasons, clinical investigation of a proposed trigger virus should control for relevant risk genes such as HLA in the study design or analysis.

If even a small subset of MS cases is associated with LCMV infection, further exploration of this subset may enhance our understanding of autoimmunity and provide new options for therapeutic interventions. We urge clinical investigators to consider the potential benefits of exploring LCMV seroprevalence among RRMS patients.

## Author contributions

The author confirms being the sole contributor of this work and has approved it for publication.

## Footnotes

1 See Genbank (https://www.ncbi.nlm.nih.gov/nuccore) accession numbers EF164923, EU136038, FJ607028, FJ607030, FJ607031, FJ607035, F607038, JN687949, JN872495, and M22138.
